# Advanced Cu chemical displacement technique for SiO_2_-based electrochemical metallization ReRAM application

**DOI:** 10.1186/1556-276X-9-592

**Published:** 2014-10-28

**Authors:** Fun-Tat Chin, Yu-Hsien Lin, Hsin-Chiang You, Wen-Luh Yang, Li-Min Lin, Yu-Ping Hsiao, Chum-Min Ko, Tien-Sheng Chao

**Affiliations:** 1 Ph.D. Program of Electrical and Communications Engineering, Feng Chia University, No. 100 Wenhwa Rd., Seatwen, Taichung 40724, Taiwan; 2 Department of Electronic Engineering, National United University, 1, Lienda, Miaoli 36003, Taiwan; 3 Department of Electronic Engineering, National Chin Yi University of Technology, No. 57, Sec. 2, Zhongshan Rd., Taiping Dist., Taichung 41170, Taiwan; 4 Department of Electronic Engineering, Feng Chia University, No. 100 Wenhwa Rd., Seatwen, Taichung 40724, Taiwan; 5 Department of Electrophysics, National Chiao Tung University, 1001 University Road, Hsinchu 30010, Taiwan

**Keywords:** Cu CDT, SiO_2_, ECM, ReRAM

## Abstract

This study investigates an advanced copper (Cu) chemical displacement technique (CDT) with varying the chemical displacement time for fabricating Cu/SiO_2_-stacked resistive random-access memory (ReRAM). Compared with other Cu deposition methods, this CDT easily controls the interface of the Cu-insulator, the switching layer thickness, and the immunity of the Cu etching process, assisting the 1-transistor-1-ReRAM (1T-1R) structure and system-on-chip integration. The modulated shape of the Cu-SiO_2_ interface and the thickness of the SiO_2_ layer obtained by CDT-based Cu deposition on SiO_2_ were confirmed by scanning electron microscopy and atomic force microscopy. The CDT-fabricated Cu/SiO_2_-stacked ReRAM exhibited lower operation voltages and more stable data retention characteristics than the control Cu/SiO_2_-stacked sample. As the Cu CDT processing time increased, the forming and set voltages of the CDT-fabricated Cu/SiO_2_-stacked ReRAM decreased. Conversely, decreasing the processing time reduced the on-state current and reset voltage while increasing the endurance switching cycle time. Therefore, the switching characteristics were easily modulated by Cu CDT, yielding a high performance electrochemical metallization (ECM)-type ReRAM.

## Background

Resistive random access memory (ReRAM) is a promising candidate for next-generation nonvolatile memory because of its simple cell structure (metal/insulator/metal), good shrinking capability, and low power consumption [1,2]. The binary oxides, such as NiO, HfO_2_, Cu-doped ZrO_2_, and Cu-doped SiO_2_, have been studied as switching layers in ReRAM applications [2-5]. The resistive switching mechanism in ReRAM has been suggested to form and rupture conductive filaments by anionic or cationic migration within the switching layer [6-10]. Anionic migration is generated by oxygen vacancy defects within the switching layer [6]; cationic migration arises by oxidation of an active electrode (such as Ag and Cu metal) [7-10]. The release of mobile cations from an active electrode into the switching layer is known as electrochemical metallization (ECM), and ReRAMs so produced are called (ECM)-type ReRAMs [10]. Recently, Cu metal and SiO_2_ materials have been widely used in ECM-type ReRAMs because they are compatible with CMOS back-end technology [11,12]. The resistive switching behavior of Cu/SiO_2_-stacked ReRAM devices suggests that Cu metallic filaments are formed and ruptured inside the SiO_2_ switching layer [11,12]. The Cu in Cu/insulator-stacked ReRAMs has been deposited by several methods such as sputtering and thermal evaporation [12,13]. However, these Cu deposition methods cannot easily control the etching process for Cu patterning nor modulate the interface between the Cu and the switching layer. Therefore, this study presents a Cu chemical displacement technique (CDT) for fabricating Cu/SiO_2_-stacked ReRAM devices in nonvolatile memory applications. Cu interconnects have already been fabricated by Cu CTD [14]. The simple Cu CDT process overcomes the difficulty of etching Cu into the required patterns and enables easy modulation of the interface between Cu and SiO_2_ during Cu deposition.

In this work, the switching characteristics of CDT-fabricated Cu/SiO_2_-stacked ReRAM devices were investigated by varying the Cu CDT processing time. The resistive switching characteristics of the CDT-fabricated Cu/SiO_2_-stacked ReRAM devices, namely, the I-V switching, cell-to-cell distributions of set/reset voltages, and data retention characteristics were measured and compared with those of a conventional Cu/SiO_2_-stacked sample.

## Methods

**Figure 1:**
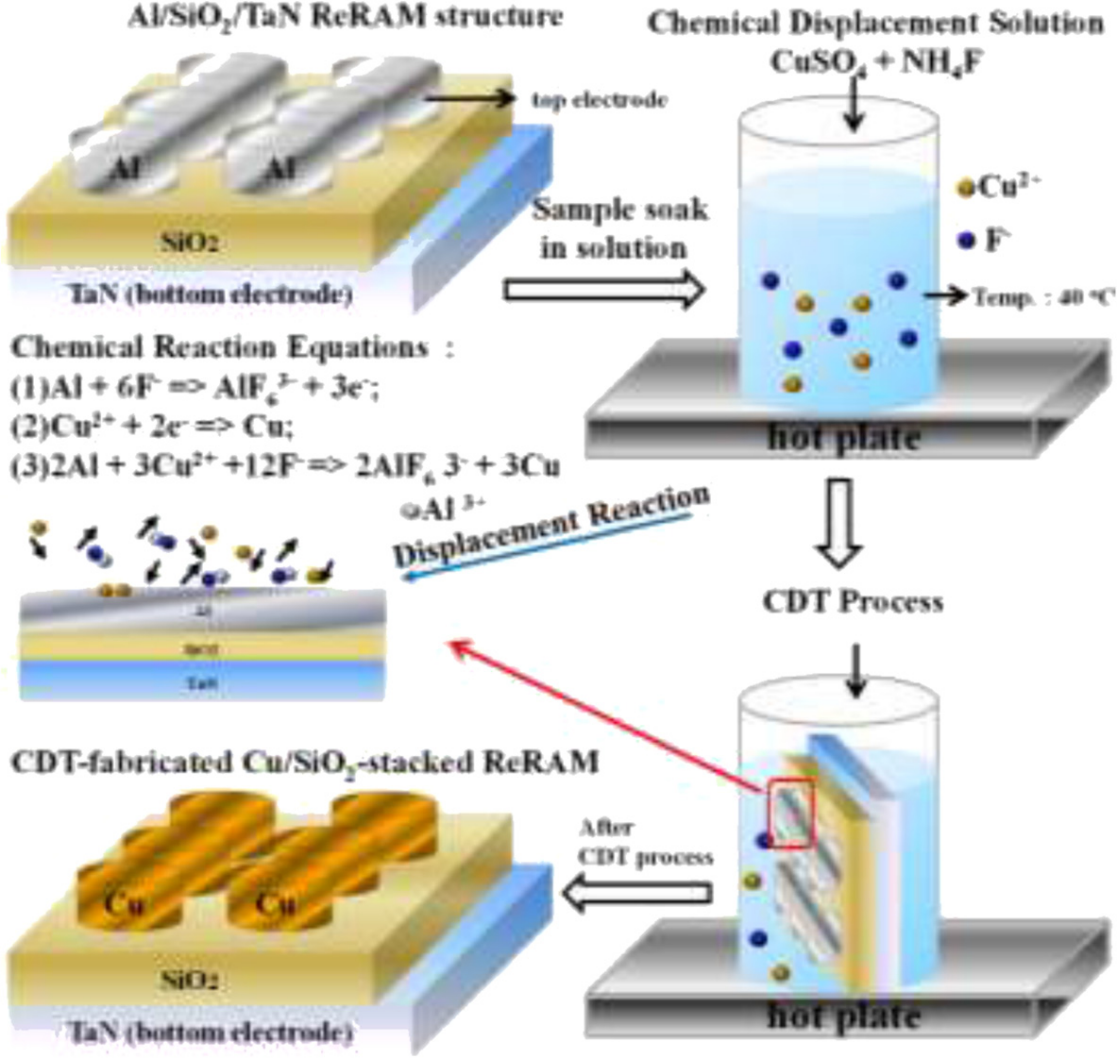
**The schematics to introduce the Cu chemical displacement technique (CDT) to fabricate Cu/SiO**_**2**_**-stacked ReRAM.** The temperature of Cu CDT process at 40°C.

A schematic of the Cu chemical displacement technique (CDT) procedure for fabricating Cu/SiO_2_-stacked ReRAM is illustrated in Figure 1. After RCA cleaning, a thermal oxide was grown on the silicon substrate to form an isolation layer, and a TaN metal film (thickness = 200 nm) was deposited on the prepared SiO_2_/Si-sub as the bottom electrode. Second, a switching layer of 20-nm-thick TEOS oxide (SiO_2_) was deposited by plasma-enhanced chemical vapor deposition. Third, a 100-nm-thick Al metal film was deposited through a metal mask using an e-beam evaporator to form the displacement layer. The resulting structure is the Al/SiO_2_/TaN ReRAM device. The completed Al/SiO_2_-stacked samples were soaked in a chemical solution (0.02 M CuSO_4_ · 5H_2_O and 0.22 M NH_4_F) for Cu CDT processing. Cu displacement occurs by the following half reactions: (1) Al +6F^−^ = > AlF_6_3^−^ + 3e^−^; (2) Cu^2+^ + 2e^−^ = > Cu; giving a total reaction of (3) 2Al + 3Cu^2+^ + 12F^−^ = > 2AlF6^3−^ + 3Cu. The displacement time was varied as 60, 65, and 70 s at 40°C. After completion of the Cu CDT process, the samples were cleaned in deionized water and blow-dried with a N_2_ gun. Finally, the samples were placed on a hot plate and baked at 80°C for 10 min to remove moisture. The dried samples were the completed CDT-induced Cu/SiO_2_/TaN ReRAMs. For comparing the resistive switching characteristics, a control Cu/SiO_2_-stacked sample was also prepared. The top Cu electrode of the control sample was deposited by a thermal evaporator. The electrical switching properties associated with I-V switching, cell-to-cell distributions of set/reset voltages, data retention, and endurance characteristics were measured for analyzing by a Keithley 4200 system (Keithley Instruments Inc., Cleveland, OH, USA).

## Results and discussion

**Figure 2:**
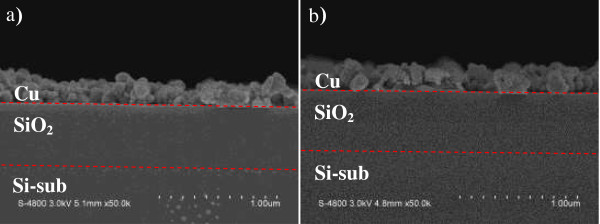
SEM image of the displacing Al with Cu (a) after 60 and (b) 70 s displacement reaction.

**Figure 3:**
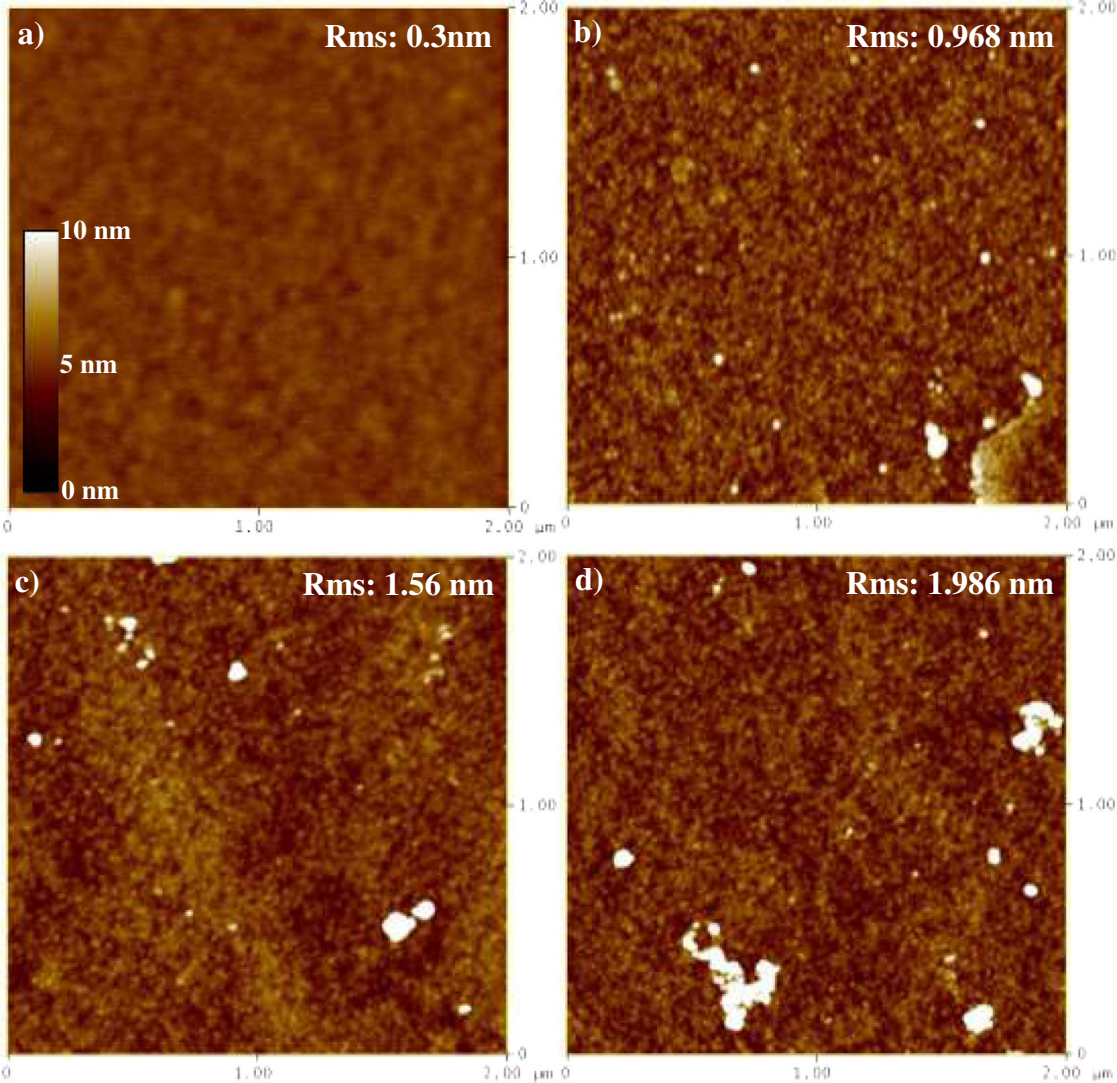
**AFM image to analyze the surface roughness of SiO**_**2**_**. (a)** Control, **(b)** CDT 60, **(c)** CDT 65, and **(d)** CDT 70 s samples.

Figure 2 shows scanning electron microscopy (SEM) images of the samples prepared by Cu CDT for 60 and 70 s. The initial SEM testing sample was an Al (100 nm)/PE-TEOS SiO_2_ (500 nm)/Si-sub structure. After Cu CDT processing, the Al metal film was completely displaced by Cu. The average thickness of the Cu in the 60 and 70-s samples was approximately 198 and 207 nm, respectively, and the thickness of the SiO_2_ film was reduced by the longer processing time (decreasing ca. 8 nm relative to the shorter time). During the longer period, fluoride ions could react with the SiO_2_, releasing extra electrons that promoted the reduction of Cu^2+^ to Cu atoms. For this reason, the Cu metal film was thicker in the 70-s sample than in the 60-s sample. In addition, the attack of F^−^ ions during the longer CDT process roughened the Cu–SiO_2_ interface. Atomic force microscopy confirmed that the SiO_2_ surface roughness increased with increasing CDT processing time (Figure 3). We observe the surface roughness of SiO_2_ film for CDT process by AFM system. For the control sample, we directly scanned the surface of fresh PECVD SiO_2_ film. As a comparison, the CDT samples were fabricated by the Cu CDT process, and then removed the Cu metal film to observe the revealed SiO_2_. The results of surface roughness for the SiO_2_ film were 0.3, 0.968, 1.56 and 1.986 nm for the control, CDT 60, CDT 65, and CDT 70 s, respectively, as shown in Figure 3. The Cu metal in samples fabricated by Cu CDT was removed by wet etching methods, revealing that the shape of the interface could be controlled by CDT.

**Figure 4:**
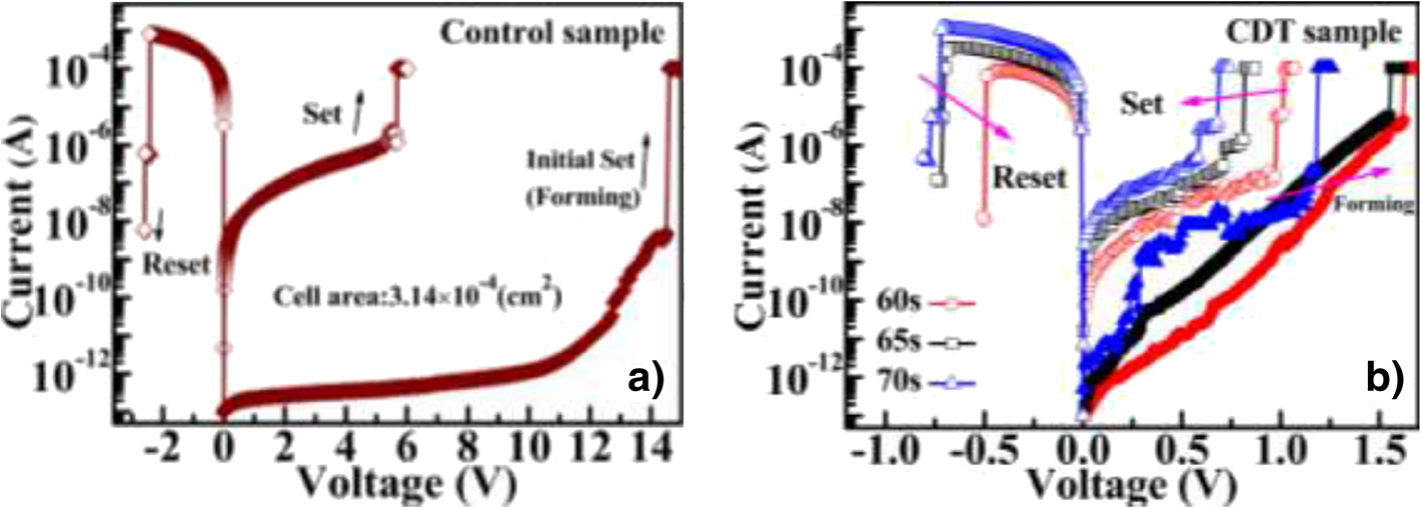
**Typical bipolar I-V resistive switching characteristics of (a) control and (b) CDT-fabricated Cu/SiO**_**2**_**-stacked (CDT sample) ReRAM.**

Figure 4 shows the typical bipolar I-V resistive switching characteristics of control sample and CDT samples. In the fresh devices, a so-called 'forming process' in which the Cu electrode is subjected to a large positive voltage bias was required for generating resistive switching behavior. The initial high resistance state (HRS) of the devices was then switched to a low resistance state (LRS). Next, the switching characteristics of the devices were repeatedly executed by a DC sweep voltage that cyclically turned the LRS on (set process) and the HRS off (reset process) by applying positive and negative voltage biases, respectively. When applying the positive voltage bias in the forming and set processes, the current was restricted to 100 μA to prevent device hard-breakdown. The voltages of the forming, set, and reset operations were found to be smaller in the CDT-fabricated samples than in the control samples. Because both control and CDT samples are ECM-type ReRAMs, their resistive switching mechanisms are expected to arise from the formation and rupture of Cu conductive filaments. In the CDT samples, the roughness interface appending effect imposed by the CDT processing could enhance the local electric field. Besides, the CDT-fabricated Cu forms different Cu structure (loose structure). The different Cu structure could also be oxidized easily and result in fast drifting into SiO_2_[12]. Based on these reasons, the consequent rapid dissolution of Cu ions would hasten the formation and rupture of Cu conductive filaments. Thus, the CDT samples obtained lower operation voltages than the control sample. In addition, the forming and set operation voltages of the CDT-fabricated samples decreased with increasing CDT processing time. This result is attributed to the reduction of SiO_2_ thickness, since each CDT-fabricated sample shows similar electric field for forming and set operation. By contrast, the reset operation voltage and the on-state current (LRS) of the CDT samples decreased as the CDT processing time decreased. The reduced on-state current was implied by the altered thickness of the Cu conductive filaments [12]. Thin Cu conductive filaments would lower the on-state current, and therefore the reset operation voltage, in CDT samples fabricated over short processing times.

**Figure 5:**
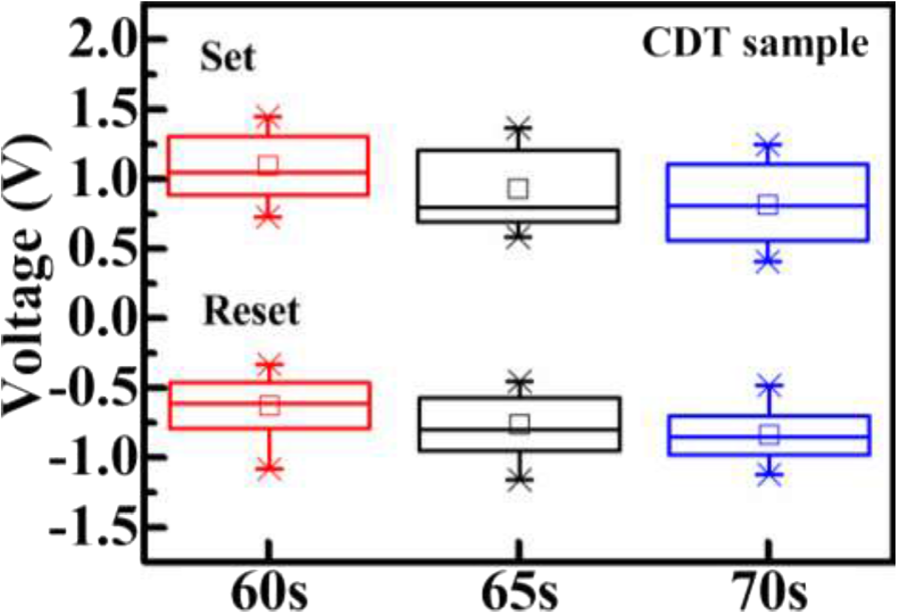
**Cell-to-cell distributions of switching voltages in CDT-fabricated Cu/SiO**_**2**_**-stacked (CDT sample) ReRAM.** Box plot statistics showing the distribution of set and reset voltages.

The cell-to-cell distributions of switching voltages in the CDT samples are illustrated in Figure 5. Twenty devices were measured to plot the set and reset voltages. The sample fabricated over a short period demonstrated a more uniform set operation voltage. This is attributed to the thin Cu conductive filaments in the SiO_2_ switching layer. The switching gap area is smaller in thin Cu conductive filaments than in their thick counterparts [12]. During the set process, the Cu ions are easily guided along the direction of the electric field in an area of small switching gap. This phenomenon explains the enhanced uniformity of the set operation voltage distribution in CDT samples fabricated over shorter times.

**Figure 6:**
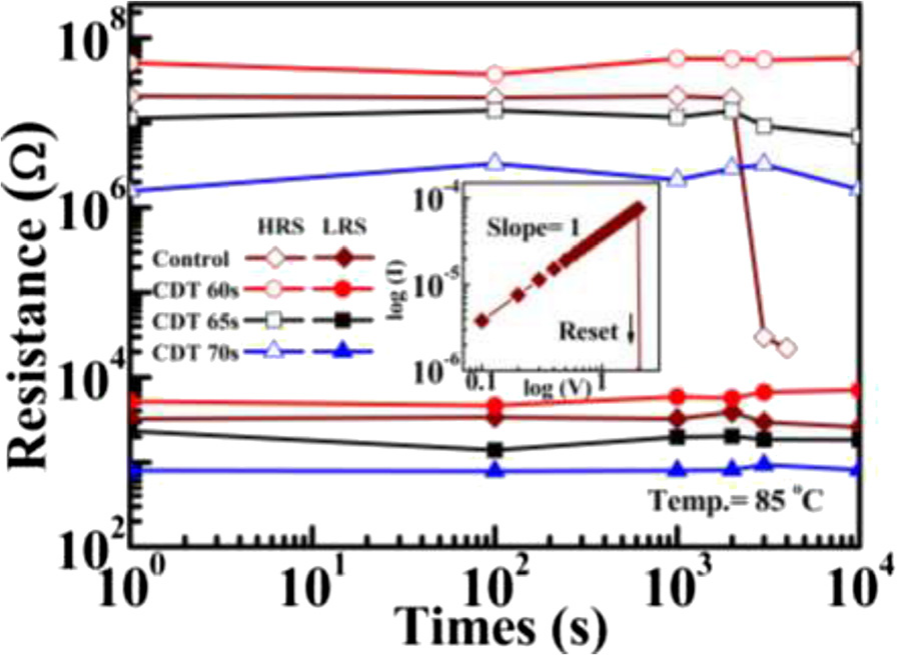
**Date retention characteristics of control and CDT-fabricated Cu/SiO**_**2**_**-stacked (CDT sample) ReRAM at 85°C testing.** The inset of the figure showing ohmic conduction in the control sample after HRS degenerate to LRS.

**Figure 7:**
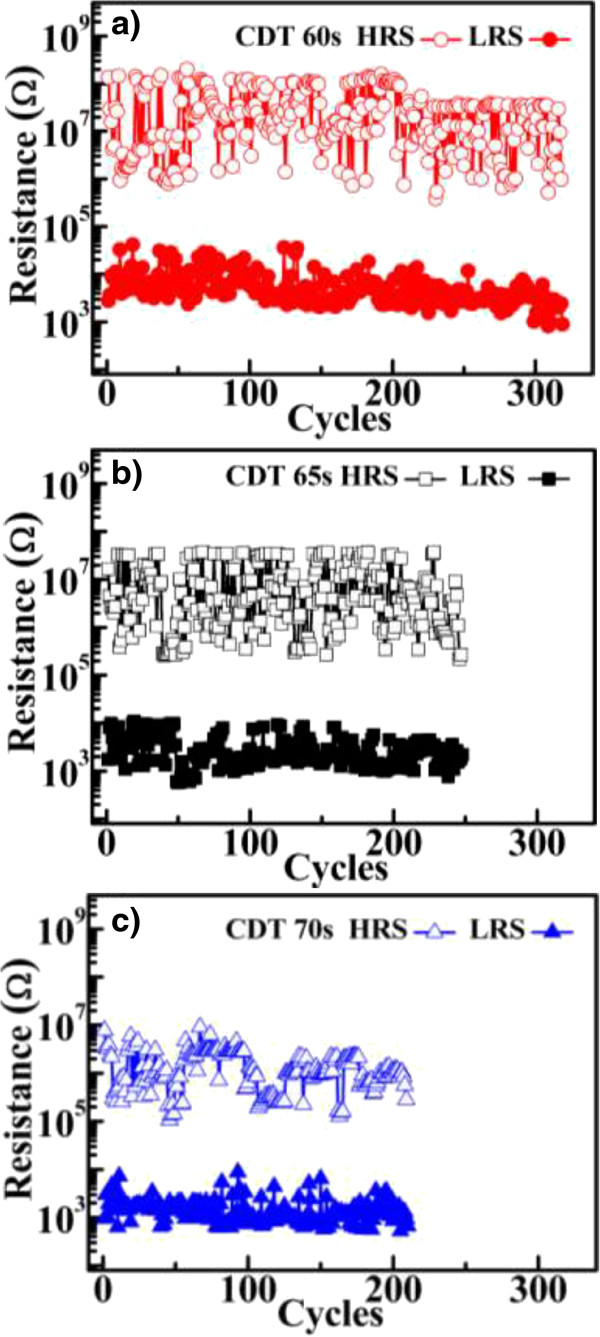
**Endurance switching cycles characteristic. (a)** CDT 60, **(b)** CDT 65, and **(c)** CDT 70 s Cu/SiO_2_-stacked (CDT sample) ReRAM. The read out voltage is 0.1 V.

Next, the effect of thermal stress on the control and CDT samples was investigated. Figure 6 shows the data retention properties of samples exposed to high temperature (85°C). The DC sweep was first turned on and off, and the CDT samples were measured at 0.1 V readout. The HRS characteristics revealed a shorter data retention time in the control samples than in the CDT-fabricated samples. Subsequent to HRS failure, the control sample could be reset to turn off the HRS, as shown in Figure 6 (inset). Post HRS failure, the fitting results of current conduction mechanism in the control sample is ohmic conduction. This implies that HRS failure occurs by the reconnection of the Cu conductive filaments [8,12]. In HRS under high thermal stress, the Cu residue in the switching layer could diffuse from high to low concentration regions and reconnect to the Cu conductive filaments induced by readout voltage stress. The CDT samples also exhibited more stable HRS characteristics than the control sample. This result is attributable to the low forming operation voltage of the CDT samples, which limits the concentration of Cu atoms entering the SiO_2_ switching layer. Consequently, the probability that Cu conductive filaments reconnect remains small. In addition, the LRS property is reportedly related to the size of the Cu conductive filaments [15]. Thin Cu conductive filaments are easily ruptured by baking at high temperatures because the metal ions readily migrate [15]. However, the LRS property of samples fabricated at short CDT processing times was negligibly degraded under high-temperature testing. This demonstrates the strong reliability of the CDT samples and highlights their potential in nonvolatile resistive switching memory applications. The endurance switching cycle characteristics of the CDT samples are shown in Figure 7. For all samples, these tests were performed using DC sweep cycles and a readout voltage of 0.1 V. The endurance switching cycle times of the CDT samples increased with decreasing CDT processing time. Failure of the endurance switching cycle is attributable to the Joule heating effect [16]. Repeated application of set/reset ultimately destroys the switching layer gap between the electrode and the Cu conductive filaments [16]. The increased endurance period of the switching cycle at shorter CDT processing times may be ascribed to the thin Cu conductive filaments formed in the switching layer. Because the operating current and reset voltage were reduced in the thin filaments, the joule heating effect was lowered and the switching layer gap was more likely preserved. Therefore, the Cu CDT process can achieve Cu/insulator-stacked devices with highly efficient and reliable switching characteristics for use in high-performance ECM-type ReRAMs.

## Conclusions

We proposed a CDT to fabricate Cu/SiO_2_-stacked ReRAMs, in which Al is displaced by Cu. Compared with conventional Cu/SiO_2_-stacked ReRAM fabrication methods, Cu CDT easily modulates the shape of the Cu/SiO_2_ interface and the thickness of the SiO_2_ switching layer during Cu deposition. The large interface roughness of the CDT samples enhanced the local electric field and therefore the speed of Cu filament formation. Besides, the Cu structure difference also could hasten the dissolution of Cu ions and result in fast drifting into SiO_2_. Thus, the CDT samples exhibited lower operation voltages and more stable data retention characteristics than the control sample. Furthermore, as the processing time decreased, the on-state currents and reset voltages of the CDT samples decreased, whereas their endurance switching cycle times increased. These results indicate a relationship between the switching characteristics and the shape of the interface; therefore, the switching characteristics can be easily controlled by the Cu CDT process. Moreover, Cu CDT can avoid the Cu etching problem and is compatible with current IC technology. Using Cu CDT, manufacturers cannot only ensure immunity against Cu etching but can also control the Cu-SiO_2_ interface and the thickness of the switching layer to realize high-performance 1-diode-1-ReRAM (1D-1R), 1T-1R, or systems-on-chip integration.

## Abbreviations

Al: aluminum; CDT: chemical displacement technique; Cu: copper; ECM: electrochemical metallization; HRS: high resistance state; LRS: low resistance state; ReRAM: resistive random access memory; 1D-1R: 1-diode-1-ReRAM; 1T-1R: 1-transistor-1-ReRAM.

## Competing interests

The authors declare that they have no competing interests.

## Authors’ contributions

FTC prepared the samples and electrical measurements. FTC, YHL, and WLY contributed to the design and experimental results analyzing and wrote the manuscript. TSC and HCY provide technical support to study. LML, YPH, and CMK assisted in the samples prepared. All authors read and approved the final manuscript.
